# Phenotypic and Functional Profiling of CD4 T Cell Compartment in Distinct Populations of Healthy Adults with Different Antigenic Exposure

**DOI:** 10.1371/journal.pone.0055195

**Published:** 2013-01-28

**Authors:** Sophie Roetynck, Ally Olotu, Joan Simam, Kevin Marsh, Brigitta Stockinger, Britta Urban, Jean Langhorne

**Affiliations:** 1 Division of Parasitology, MRC National Institute for Medical Research, London, United Kingdom; 2 Kenya Medical Research Institute/Wellcome Trust Research Programme, Centre for Geographic Medicine Research, Kilifi, Kenya; 3 Centre for Tropical Medicine, Nuffield Department of Medicine, University of Oxford, Oxford, United Kingdom; 4 Division of Molecular Immunology, MRC National Institute for Medical Research, London, United Kingdom; 5 Molecular Parasitology and Immunology, Liverpool School of Tropical Medicine, Liverpool, United Kingdom; University of Massachusetts Medical School, United States of America

## Abstract

**Background:**

Multiparameter flow cytometry has revealed extensive phenotypic and functional heterogeneity of CD4 T cell responses in mice and humans, emphasizing the importance of assessing multiple aspects of the immune response in correlation with infection or vaccination outcome. The aim of this study was to establish and validate reliable and feasible flow cytometry assays, which will allow us to characterize CD4 T cell population in humans in field studies more fully.

**Methodology/Principal Findings:**

We developed polychromatic flow cytometry antibody panels for immunophenotyping the major CD4 T cell subsets as well as broadly characterizing the functional profiles of the CD4 T cells in peripheral blood. We then validated these assays by conducting a pilot study comparing CD4 T cell responses in distinct populations of healthy adults living in either rural or urban Kenya. This study revealed that the expression profile of CD4 T cell activation and memory markers differed significantly between African and European donors but was similar amongst African individuals from either rural or urban areas. Adults from rural Kenya had, however, higher frequencies and greater polyfunctionality among cytokine producing CD4 T cells compared to both urban populations, particularly for “Th1” type of response. Finally, endemic exposure to malaria in rural Kenya may have influenced the expansion of few discrete CD4 T cell populations with specific functional signatures.

**Conclusion/Significance:**

These findings suggest that environmentally driven T cell activation does not drive the dysfunction of CD4 T cells but is rather associated with greater magnitude and quality of CD4 T cell response, indicating that the level or type of microbial exposure and antigenic experience may influence and shape the functionality of CD4 T cell compartment. Our data confirm that it is possible and mandatory to assess multiple functional attributes of CD4 T cell response in the context of infection.

## Introduction

CD4 T cells are a central component of the immune response to a wide variety of pathogens. Nevertheless, in most infections, the precise mechanisms of CD4 T cell mediated protection remain uncertain. Besides their mandatory role in inducing humoral immunity, CD4 T cells also produce cytokines, which control infection and regulate responses to limit pathology, and they seem to be required for the optimization and maintenance of CD8 T cell memory [1]. On the other hand, there are examples where they can drive pathogenesis [2], [3] and, despite extensive efforts, in most instances, such as in malaria, it is still unclear under which circumstances CD4 T cells mediate either protective immunity or immunopathology.

The CD4 T cell memory pool comprises a heterogeneous population of antigen (Ag)-experienced cells extremely diverse, not only in terms of Ag specificity, but also surface phenotype, effector function and memory potential (reviewed in [4], [5], [6]). These phenotypic and functional properties of CD4 T cell responses may be modulated by a variety of factors including innate immune responses, microenvironment, time to Ag exposure as well as Ag load, availability and persistence [7], [8], [9]. Until recently, many studies of CD4 T cell responses focused only on one or a limited number of T cell attributes, such as magnitude of the response and expression of one or a few signature cytokines or cell surface markers, often showing little correlation with disease status or immunity. This is not surprising given the recent findings showing flexibility in CD4 T cell commitment as well as plasticity in their cytokine secretion profiles (reviewed in [10]) and the existence of memory cells with overlapping cytokine patterns, which do not conform to T helper classification [11], [12]. In addition, recent studies of Ag-specific T cell responses to vaccines, or after certain infections (reviewed in [13], [14]), have revealed an enormous phenotypic heterogeneity and functional diversity within the CD4 T cell population, emphasizing the importance of the quality (i.e. the combination of different functions at a single cell or cell population level) of a T cell response, as opposed to its simple magnitude or response of a single population, in correlation with clinical outcome. Together, these studies have demonstrated that the simultaneous assessment of multiple attributes of T cell function is necessary for the prediction of the outcome of infection or vaccination.

Multiparameter flow cytometry (FC) [15] allows the simultaneous assessment of magnitude, phenotype and multiple functional characteristics of CD4 T cells, including their differential expression of surface markers defining distinct populations at different stages of differentiation [4], [16], their cytokine production as well as their homing receptors profiles [4], [17]. The aim of this study was therefore to establish reliable and feasible assays, which will allow us to characterize more fully CD4 T cell population in humans in field studies.

We have designed antibody (Ab) panels for polychromatic FC analysis of chemokine receptor and activation marker expression patterns as well as cytokine profiles. In a pilot (“proof-of principle”) study, we analysed CD4 T cell responses of healthy adults, from a rural population of coastal Kenya, and compared with those of age and sex-matched African and European individuals, living in urban Kenya. We show that the global expression profile of CD4 T cell maturation and activation markers differed significantly between African and European healthy adults while no major differences were observed amongst African individuals from either rural or urban areas. Furthermore, we observed quite distinct patterns of cytokine production between the rural and the urban populations, with a predominance of “Th1” over “Th2” type of cytokines, a greater number of subsets expressing multiple cytokines as well as higher frequency of multifunctional CD4 T cells in the rural community, suggesting that environmental factors and/or antigenic experience can affect the functional signature of CD4 T cell response. Although very little correlation was found between malaria exposure and the functional attributes of the global response in blood, endemic exposure to malaria may contribute to the expansion of a few discrete CD4 T cell populations with specific functional profiles as a possible mechanism to prevent immunopathology associated with the infection. Collectively, our data suggest that environmentally driven T cell activation in rural Kenya does not lead to the dysfunction of CD4 T cell population but is rather associated with a CD4 T cell cytokine response of higher magnitude and greater polyfunctionality, suggesting that the level and/or the type of microbial exposure and antigenic experience may influence and shape the functionality of CD4 T cell compartment.

## Results and Discussion

### Characteristics of the Study Population

CD4 T cell responses from 25 healthy adults from rural Kenya (rural African donors, RA) were analysed and compared to those from age and sex-matched urban populations of African (urban African donors, UA, n = 8) as well as European (urban European donors, UE, n = 8) individuals. The rural population comes from villages located in the Junju sublocation of Kilifi district [18], while the urban populations live in the urban area of Kilifi town, in coastal Kenya. European donors grew up in Europe but were currently living in Kilifi town at the time of the study.

### The Overall Profile of CD4 T cell Differentiation is Significantly Different between African and European Individuals but Similar between Rural and Urban Populations of Healthy African Adults

Upon activation, naïve CD4 T cells develop into effector cells, which gain progressively functionality with differentiation [4], [13], [19]. During this process, they give rise to central memory (T_CM_) and effector memory cells (T_EM_) that will expand rapidly upon secondary challenge [19], [20]. It is possible to define stages of T cell activation and maturation by differential expression of cell surface markers, such as CD45RA, CCR7, CD27 and CD28 [4], [16], [19]. While naïve CD4 T cells express all four markers, they progressively lose surface expression of these molecules as they undergo activation and differentiation, such that T_CM_, or early/middle effectors, are CCR7^+^ CD27^+^ CD28^+/−^ CD45RA^−^ and T_EM_, or middle/late effectors, are CCR7^−^ CD27^+^ CD28^+/−^ CD45RA^+/−^ [4], [16], [19].

**Figure 1:**
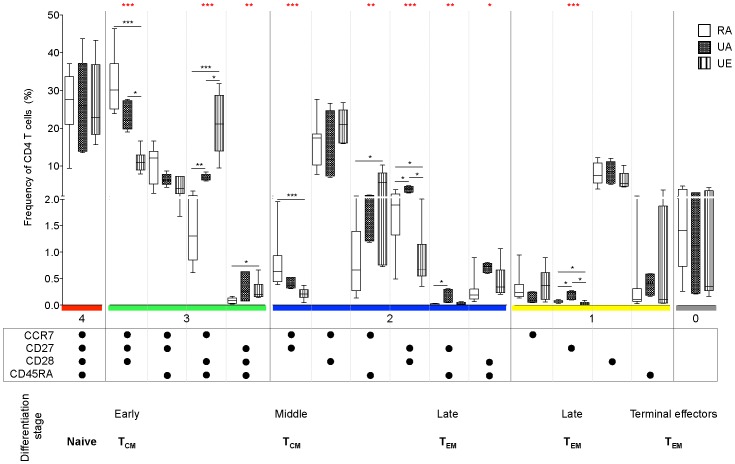
CD4 T cell differentiation and activation profile significantly differs between African and European donors but is similar between African individuals from either rural or urban Kenya. The differential expression of CCR7, CD27, CD28 and CD45RA by CD4 T cells was analysed by Boolean gating. Each phenotype (defined by a specific combination of markers) is shown under each subset. Each dot denotes the expression of each marker indicated on the bottom left. The differentiation stage is specified when a specific phenotype has been ascribed to a particular stage 4,13,19]. T_CM_ and T_EM_ refer to central memory and effector memory cells, respectively. CD4 T cell subsets were then ordered according to the number of markers they express specified by the horizontal bars of different colours showing these combinations of 4, 3, 2, 1 or 0 marker. The frequency of each subset is represented as a percentage of the total CD4 T cells (medians and 95% confidence intervals are shown for each group) and was compared between the three groups (rural African (RA, n = 25), urban African (UA, n = 8), and urban European donors (UE, n = 8)). Differences in CD4 T cell phenotypic profile among groups were tested using Kruskal-Wallis test and where significance was obtained, nonparametric Mann-Whitney U test was used for pair-wise analysis of the differences between groups. Statistically significant *P*-values (<0.05) are indicated by an asterisk (in red for Kruskal-Wallis or black for Mann-Whitney tests, respectively). *indicates P<0.05, **P≤0.01 and ***P≤0.001.

Studies on chronic viral infections in humans suggest that sustained and chronic antigenic stimulation can result however in progressive loss of both their ability to produce cytokines and their memory potential, leading to pro-apoptotic terminally differentiated CD4 T cells, defined as CCR7^−^ CD27^−^ CD28^−^ CD45RA^+/−^ [9], [14], [21]. Since the three different groups of healthy volunteers (rural and urban African and urban European donors) are likely to have been exposed to both quantitatively and qualitatively distinct microbial environments and have had different antigenic experience, we examined whether this might be reflected on the distribution of CD4 T cell maturation and activation phenotypes. We therefore compared the cell surface expression of CD45RA, CCR7, CD27 and CD28 (panel Table S1) on CD4 T cells in PBMC. Boolean gating analysis identified 16 subpopulations expressing each possible combination of markers within the CD4 T cell population (Figure 1, gating strategy is shown in Figure S1). In all groups, approximately 26% of CD4 T cells could be defined as naïve as they expressed all 4 markers, in agreement with previous studies on healthy individuals in the United States [22] and Burkina Faso [23].

Remarkably, all the 16 possible subpopulations were observed in all the donors, confirming the great heterogeneity of CD4 T cells with respect to expression of these markers in PBMC. The well-described CD4 T cell populations of T_CM_ as well as T_EM_ cells were present, but also all the intermediate phenotypes, which can not as yet be ascribed to a specific subpopulation or to a functionally unique subset [4], [16]. Across group comparisons using Kruskal-Wallis test showed that, out of these 16 subsets, the frequency of 9 subpopulations was significantly different between individuals from rural Kenya and the two urban populations of volunteers. Four phenotypes (CCR7^+^ CD27^−^ CD28^+^ CD45RA^+^; CCR7^−^ CD27^+^ CD28^+^ CD45RA^−^; CCR7^−^ CD27^+^ CD28^−^ CD45RA^+^ and CCR7^−^ CD27^+^ CD28^−^ CD45RA^−^, respectively) were significantly less frequent in the rural population when compared to the urban African population (P = 0.007; P = 0.01; P = 0.01 and P = 0.01, respectively). Significant differences were, for the majority, observed between rural African and urban European donors. However, they were also, for most of them, observed between the two urban populations, suggesting that differences in CD4 T cell subsets between African and European individuals may be due to either genetic or other environmental factors rather than their current microbial exposure.

The frequency of CD4 T cells exhibiting the CCR7^−^ CD27^−^ CD28^−^ CD45RA^+/−^ phenotype, defined as terminally differentiated effector memory cells [4], [13], [16], was very low in all donors (less than 2%) and there was no significant difference between rural and urban groups of individuals. Furthermore, differentiation towards a terminal effector stage has been associated with a decrease in CD28 expression by CD4 T cells and the appearance of CD28^−^ late effector CD4 T cells [4]. No significant differences were found in the frequency of CD28 positive and CD28 negative CD4 T cells between groups (data not shown), further confirming that the rural population does not have a greater frequency of CD4 T cells with phenotypic characteristics of terminal differentiation associated with T cell “exhaustion” or dysfunction [24].

In summary, the differentiation and activation marker expression profiles were found comparable between African individuals from rural and urban Kenya. This is in complete agreement with a previous study showing no detectable qualitative differences in the distribution of lymphocyte subsets in HIV negative healthy adults living in either a rural or an urban area in Ethiopia [25]. Collectively, our data suggest that environmentally triggered T cell activation in rural Kenya does not seem to drive the general exhaustion of CD4 T cells, despite significantly higher levels of reported chronic and acute conditions in the rural as compared with the urban setting [26]. Earlier studies, using only CD45RA and CCR7 or CD27 as markers, found more memory and fewer naïve T cells in PBMC of African compared to European donors [23], [25], [27]. We did not observe similar skewed distribution of CD4 T cell phenotypes, when 4 markers were used to distinguish CD4 T cell types. However, our analysis did reveal more subtle differences between African and European individuals, mainly in the Ag-experienced CD4 T cell compartment, suggesting that the distribution pattern of T cell maturation phenotypes may not be a strictly direct surrogate parameter of environmentally driven T cell stimulation, and reflected a more complex relationship between phenotype and Ag experience.

### CD4 T Cell Cytokine Responses from Rural African Donors are of Higher Magnitude Compared to those of Urban Individuals

Upon activation, CD4 T cells tailor their response to the type of invading microbe by differentiating into T helper (Th) cells with specific effector functions, characterized by distinct profiles of gene expression and cytokine production.

To determine whether CD4 T cell pool of healthy adults living in rural and urban environments have different functional profiles, we analysed the production of cytokines characteristic of different CD4 T cell subsets, in PBMC from each group of individuals.

**Figure 2:**
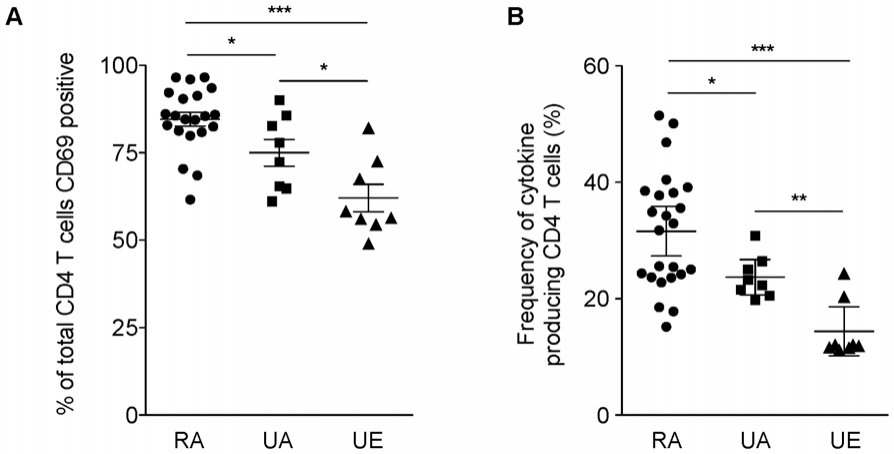
The magnitude of CD4 T cell cytokine response is higher in the rural population. CD4 T cell cytokine responses from adults living in rural Kenya (RA, n = 25) were analysed and compared to those from urban populations of African (UA, n = 8) and European (UE, n = 8) donors. The functional signatures of CD4 T cells were determined after non-specific *in vitro* stimulation with PdBU and ionomycin via the analysis of an array of functions including IFNγ, IL-2, IL-10, IL-17, TNFα, IL-21, IL-22, IL-4 and IL-9 secretion (panels 1-3 Table S1). (A) The total frequency of CD69 positive CD4 T cells following stimulation was expressed as a percentage of total CD4 T cells. (B) The total frequency of CD4 T cells expressing at least one cytokine was expressed as a percentage of CD4 T cells (%). Horizontal bars indicate the mean (95% confidence intervals are represented) for each group. Nonparametric Mann-Whitney U test was used to analyse differences in the T cell responses between groups. Statistically significant *P*-values (< 0.05) are indicated by an asterisk (P<0.05 *; P≤0.01 **; P≤0.001 ***).

CD4 T cells from the rural African individuals were the most responsive to *ex vivo* stimulation with mitogen as both the percentage of CD4 T cells which had upregulated CD69 and the mean level of CD69 expression per cell (data not shown) were significantly higher when compared to those from the urban African and European donors (Figure 2A, P = 0.03 and P = 0.0003, respectively). The total frequency of CD4 T cells expressing any cytokine was significantly higher in the rural African donors compared with those from both urban African and European individuals (Figure 2B, P = 0.03 and P = 0.007 respectively) (gating strategy shown in Figure S2), with an overall lower responsiveness and decreased frequency of cytokine secreting CD4 T cells in European compared to African donors. These differences in cytokine secreting CD4 T cell population between rural and urban African communities and also between African and European donors could reflect differences in the level of microbial exposure or in Ag experience across the groups.

**Figure 3:**
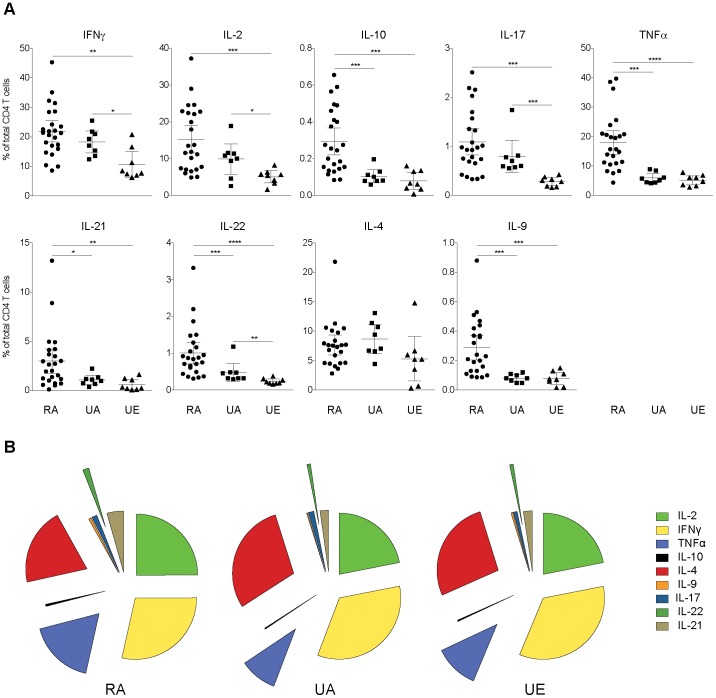
Functional analysis of CD4 T cell cytokine response in the three groups of donors. (A) The frequency of CD4 T cells positive for each cytokine (IFNγ, IL-2, IL-10, IL-17, TNFα, IL-21, IL-22, IL-4 and IL-9, respectively) is represented as a percentage of CD4 T cells (mean (%) and 95% confidence intervals are indicated) and compared between the 3 groups of donors (rural African donors, RA, n = 25; urban African donors, UA, n = 8; urban European donors, UE, n = 8). Nonparametric Mann-Whitney U test was used to analyse differences in the T cell responses between groups. An Asterisk indicates statistically significant *P*-values (< 0.05) (*P<0.05; **P≤0.01; ***P≤0.001). The pie charts in B show the relative frequency of the different cytokine responses within the overall cytokine producing CD4 T cell population. The mean frequency of CD4 T cells positive for each cytokine is represented as a fraction of the total cytokine response by CD4 T cells (%) and compared between the 3 groups of donors (RA, n = 25; UA, n = 8; UE, n = 8).

We next determined the frequency of each individual cytokine response by CD4 T cells from each study population (Figure 3A). Comparisons across the three groups using Kruskal-Wallis test revealed that, except for IL-4, the total frequency of CD4 T cells producing each cytokine differed between groups. One of the most striking differences between rural and urban populations was observed for TNFα expression. Individuals from rural Kenya had approximately 3 times more CD4 T cells producing TNFα compared to both urban groups (P = 0.0003 and <0.0001 when compared to UA and UE, respectively). This observation is of particular interest given the dual role of this inflammatory cytokine in both protection and immunopathology associated with infections [28], [29], [30].

The frequency of IL-10 producing CD4 T cells was low but significantly higher in the rural population compared to either urban group (P = 0.0007 and P = 0.0006, when compared to UA and UE, respectively). Further pair-wise comparisons showed that CD4 T cells producing all of the other cytokines, with the exception of IL-4, were significantly more frequent in the rural group compared to the European volunteers. Similarly, the frequency of CD4 T cells producing IL-21, IL-22 or IL-9 was also significantly elevated in rural African individuals when compared to urban African donors (P = 0.03, P = 0.009, P = 0.0004, respectively). There was a similar trend of higher frequencies of IFNγ, IL-2 or IL-17 producing CD4 T cells between the two groups of African donors; however, these differences did not reach significance.

Despite the differences in the magnitude of the individual cytokine responses across the groups, IL-2 and IFNγ, together with TNFα and IL-4 producing CD4 T cells represented the majority of the cytokine secreting cells in all three study groups (Figure 3B). The relative proportion of cells producing TNFα and/or IL-22 was significantly higher in CD4 T cells from rural individuals compared with that of both groups of urban volunteers (P = 0.0003 and 0.04 for TNFα, and P = 0.02 and 0.02 for IL-22, when compared to UA and UE, respectively). By contrast, the rural population had a significantly smaller fraction of IL-4 expressing CD4 T cells compared to the urban individuals (P = 0.01 and 0.04 when compared to UA and UE, respectively). Interestingly, the composition of CD4 T cell cytokine response was extremely similar between the two urban groups.

Collectively, these data show that, in general, African individuals from a rural community have a greater overall cytokine response compared to individuals from an urban area, suggesting that the magnitude of the CD4 T cell cytokine response might represent a proxy marker for environmentally driven T cell activation. In addition, despite a reported high prevalence of helminthic infection in the area [31], their CD4 T cell functional profile was dominated by “Th1” signature cytokines, suggesting that their rural environment might contain numerous pathogens triggering this type of response. These observations are in contradiction with the idea that the host immune system in developing countries is biased towards a “Th2” type of profile and that the chronic immune activation, that might occur in rural Africa, may be associated with decreased cellular responses to mitogen stimulation [27], [32], [33]. All together, this indicates that studies based on the comparison between African cases and European controls must be interpreted with caution, as European donors may not be appropriate controls when CD4 T cell responses are assessed by their single magnitude; however, they seem to represent acceptable controls when the comparison is based on the composition of the T cell cytokine response.

### Rural African Donors have a Highly Polyfunctional CD4 T Cell Pool Compared to the Urban Populations

The simultaneous measurement of a variety of cytokines enables the distinction between numerous T cell subpopulations with specific functional signatures. The delineation of T cells into distinct functional populations defines the quality of the response [13]. Recently, a series of studies have shown that the quality of a T cell response is an important parameter in defining clinical immune correlates [13], [14].

To detect the different cytokines in CD4 T cells from the three groups of donors, we used three Ab panels (panels 1–3, Table S1), measuring up to 5 cytokines simultaneously. We first analysed the patterns of cytokine positive CD4 T cells from the three groups for each Ab panel and ordered the different subsets according to the number of cytokines they simultaneously express. Using all three panels of anti-cytokine Ab, the majority of cytokine secreting CD4 T cells were expressing one or two cytokines, although subsets producing 3 or even 4 cytokines were observed (Figure S3).

**Figure 4:**
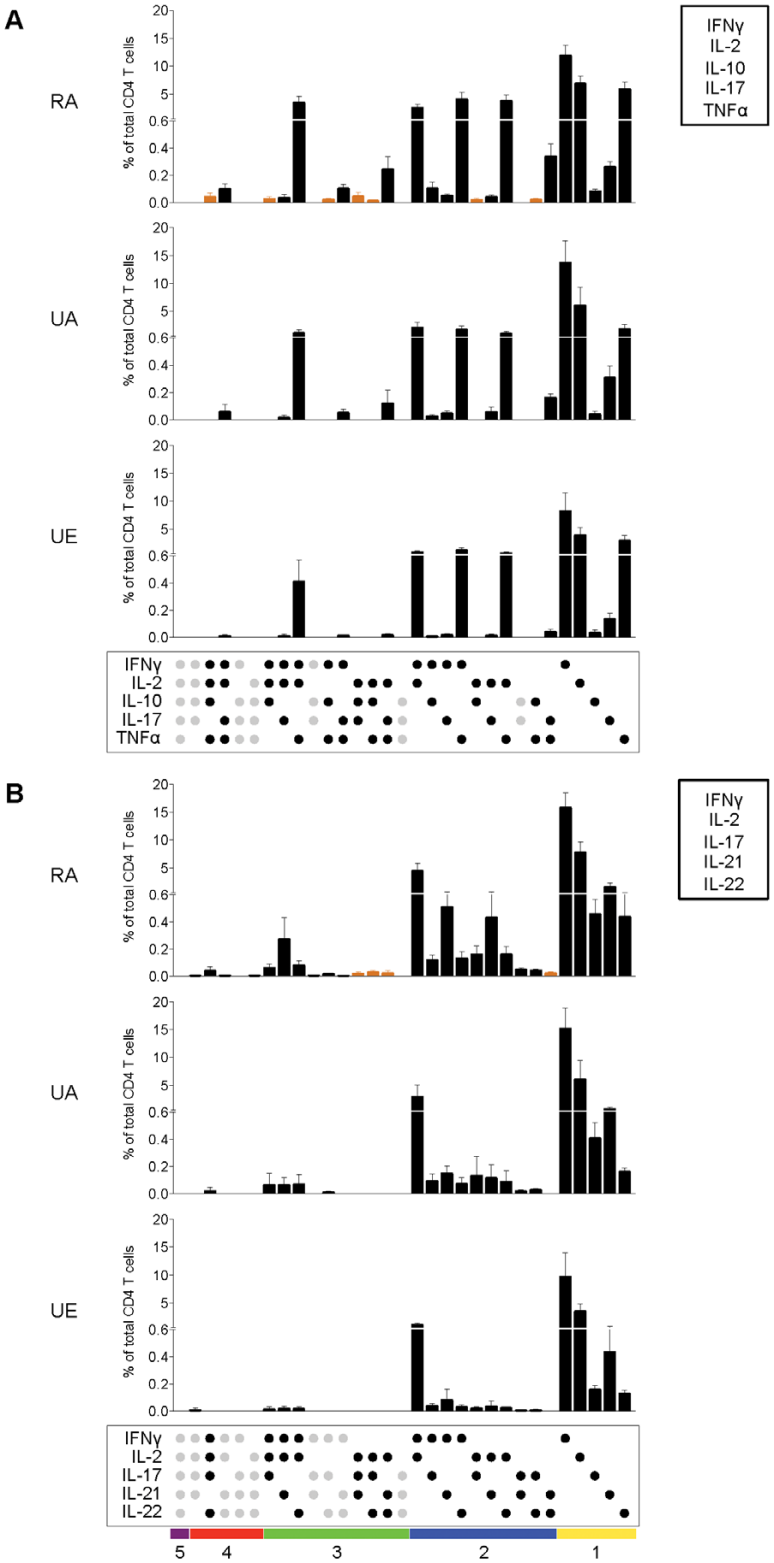
The functionality of CD4 T cell pool differs between rural and urban populations. CD4 T cell cytokine responses from rural African donors (RA, n = 25) were analysed by assessing simultaneously IFNγ, IL-2, IL-10, IL-17 and TNFα expression using panel 1 (A) or the expression of IFNγ, IL-2, IL-17, IL-21 and IL-22 using panel 2 (B) (Table S1). These responses were then compared to those from urban African (UA, n = 8) and European donors (UE, n = 8). The frequency of each CD4 T cell subset expressing the particular combination of cytokines, shown in the panel below each plot, is represented as a percentage of total CD4 T cells (mean (%) and 95% confidence intervals are indicated) for each group of donors. Each dot designates positivity for the cytokine indicated on the bottom left. Amongst the array of theoretically possible cytokine combinations, those observed are represented with black dots while those designated with grey dots were not observed. Orange bars represent the subpopulations that were observed among the CD4 T cells of the rural African donors but were completely absent in the CD4 T cells of both urban groups. Horizontal bars of different colors show these combinations of 5, 4, 3, 2 or 1 cytokine.

When IFNγ, IL-2, IL-17, IL-10 and TNFα production was simultaneously assessed using panel 1 (Table S1), 17 out of the 32 possible distinct subsets of CD4 T cells were observed in all the donors (Figure 4A). With this panel of cytokines, CD4 T cells from rural African individuals had a qualitatively different functional profile compared with the urban donors. There was more diversity among their cytokine producing CD4 T cells compared to the two urban groups, as some subsets (represented by orange bars), particularly those positive for more than 2 cytokines, were only observed within the CD4 T cells of this group. In addition, there were a significantly greater frequency of CD4 T cells from rural African donors producing 2, 3 or 4 cytokines and a decreased frequency of CD4 T cells expressing single cytokine compared to the urban donors. Among these multifunctional subsets, rural African donors had a significantly higher frequency of IFNγ/IL-2/TNFα triple producers compared to the two urban groups (P = 0.02 and P = 0.0005 when compared to UA and UE, respectively). This observation is interesting as several studies have found a correlation between this particular CD4 T cell subset and both protection following immunization and successful outcome of several infections, driving a “Th1” type of response ([34], reviewed in [13], [14]).

When IFNγ, IL-2, IL-17, IL-21 and IL-22 production by CD4 T cells was simultaneously analysed (panel 2, Table S1), single cytokine secreting CD4 T cells were again the most highly represented populations within CD4 T cells from all three groups, although different double producers and a few populations producing 3 cytokines were also present (Figure 4B). There was a similar bias towards an increase in the relative frequency of subsets co-expressing two or 3 cytokines and a decreased proportion of single cytokine-producing CD4 T cell subsets in rural individuals when compared with both urban groups (Figure S3B).

Finally, when “Th2/Th9” type of functional profiles were analysed and the production of IL-2, IL-10, IL-4 and IL-9 was simultaneously evaluated (panel 3, Table S1), there was remarkably little diversity in the functional profile of CD4 T cells subsets. The overall response was almost exclusively limited to subsets expressing a single cytokine (Figure S3C). One possible explanation is that other cytokines, such as IL-5 and IL-13, may be important to consider for the analysis of the functional profile of “Th2” type of responses. Unfortunately, it was not possible to include these mediators in our analysis, due to the lack of reagents available. Nonetheless, IL-9 and IL-4 production profiles differed significantly between the rural and urban populations of African individuals (Figure S3C). Few CD4 T cell populations expressing IL-4 and/or IL-9 in combination with other cytokines were observed and found in general more frequent in the rural African individuals when compared to the urban group of African individuals. Since “Th2” type of responses, and particularly IL-9, have been proposed to play a role in immunity against helminthic infections [35], these responses might reflect differences in exposure to this type of pathogen between the two African populations. A high prevalence of soil-transmitted helminthic infections has been reported in Coastal Kenya [36], where rural residence has been associated with higher risk of infection compared to urban setting [37]. In addition, Bejon *et al* have reported a prevalence of 25% of gastrointestinal helminth infection and 50% of eosinophilia in young children in the Junju area [31]. Further studies, measuring for instance IgE or eosinophilia levels in these individuals are therefore required to explore the possible association of these responses with exposure to helminthic infection in this area.

No major differences were observed in the functional profile of CD4 T cell responses between the two urban groups, further indicating that European donors may be acceptable controls for studies of CD4 T cell cytokine responses from African donors based on the quality of the response.

**Figure 5:**
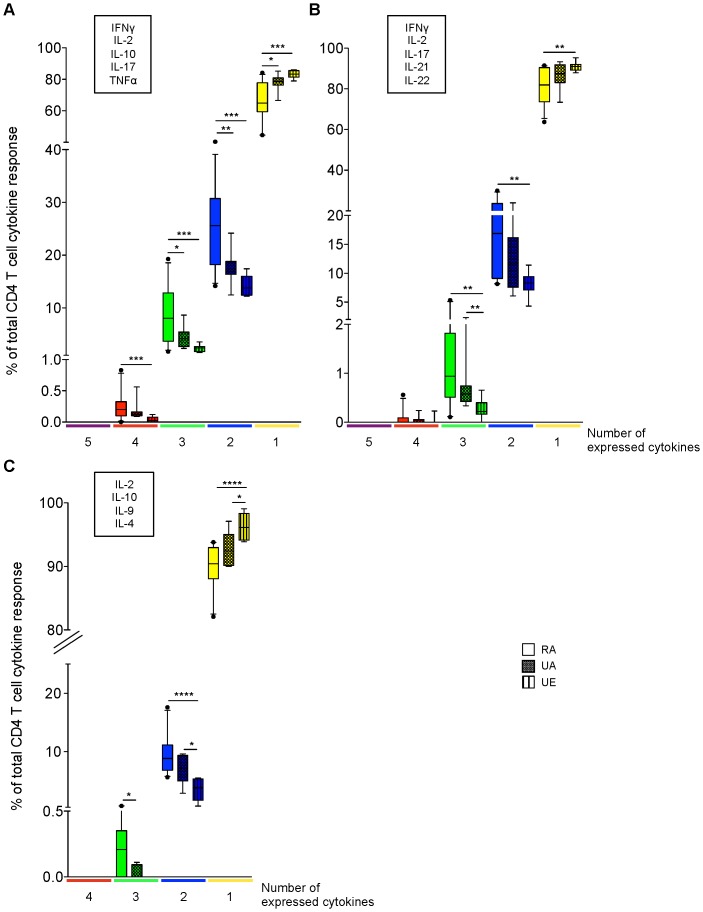
Rural individuals have a higher frequency of multifunctional CD4 T cells compared to urban donors. The different functional subsets of CD4 T cells (represented in Figure S3) were grouped by colour according to the number of cytokines they simultaneously express (the subsets expressing 5 cytokines are in purple, 4 in red, 3 in green, 2 in blue or 1 in yellow). For the functional profiles to be compared between the groups irrespective of any differences in frequency, the responses were normalized. The total number of CD4 T cells positive for at least one cytokine was considered as 100%. The frequency within the CD4 T cell population of the single, double, triple, quadruple and quintuple producers was calculated as a fraction of the total cytokine response by CD4 T cells and expressed as a percentage and is shown for each group of donors (rural African (RA, n = 25), urban African (UA, n = 8) and urban European donors (UE, n = 8)) (medians (%) and 95% confidence intervals are indicated for each group). A shows the results for panel 1; B and C represent the data obtained with panel 2 and 3 (Table S1), respectively. The cytokines measured simultaneously using the different Ab panels are indicated above each plot. Differences in CD4 T cell responses across groups were tested using Kruskal-Wallis test (data not shown) and where significance was obtained, Mann-Whitney U test was used for pair-wise analysis of the differences between groups. Statistically significant *P*-values (< 0.05) are indicated in (A) by an asterisk (P<0.05 *; P≤0.01 **; P≤0.001 ***).

We next expressed each individual response (i.e. each specific combination of cytokines) as a fraction of the total CD4 T cell cytokine response (Figure S3) and grouped the different functional subsets according to the number of cytokines they simultaneously express to determine the overall quality of CD4 T cell responses in each study population (Figure 5). Confirming the data in Figure 4, for all Ab panels, the majority of CD4 T cells from all the individuals were single cytokine secreting cells. However, we observed a higher fraction of multifunctional CD4 T cells (i.e. CD4 T cells expressing more than one cytokine) with panel 1 (Figure 5A) compared to the other Ab panels (Figure 5B and 5C). In addition, with this panel, significantly fewer CD4 T cells from PBMC of the rural population expressed only one cytokine, while multifunctional CD4 T cells represented a higher percentage (35% (mean)) of their total CD4 T cell cytokine response compared to the urban groups (Figure 5A). Similar trends were observed for panels 2 and 3, however they did not reach significance (Figure 5B and 5C).

This analysis indicates that CD4 T cell responses of rural African individuals are characterized by the presence of many distinct functional cell populations, particularly “Th1” type of responses, compared with those of individuals living in an urban area. Our data suggest that the extent of polyfunctionality of the response as well as the frequency of CD4 T cells co-expressing multiple cytokines may reflect the level of environmentally driven T cell stimulation.

### Influence of Endemic Exposure to Malaria Infection on the Phenotypic and Functional Attributes of CD4 T Cell Responses

One of the known differences in the pathogenic environment of the three groups of donors was the level of exposure to malaria. The rural population lives in a sublocation of Kilifi district, where malaria infection is endemic [18], while the urban donors live in Kilifi town, where there is no malaria. In line with this, none of the urban donors had detectable IgG Ab specific for *P. falciparum* (Apical Membrane Antigen (AMA)-1, Merozoite Surface Protein (MSP)-1_42_, Merozoite Surface Protein (MSP)-2 and total *P. falciparum* schizont extract (*Pf*SE)), whereas these Ab were detectable in the plasma of all rural African donors at variable levels (Figure S4), suggesting that these individuals have been exposed to malaria [38] and have developed clinical immunity to the infection [39], [40], [41]. In addition, malaria exposure indexes [42] were estimated for a period of time starting in 2009 until 2010 and confirmed that all donors were likely to have been exposed to the infection during the last 2 years preceding this cross-sectional survey (data not shown). However, none of the exposed volunteers was infected with *P. falciparum* at the time of sampling as confirmed by both negative blood smears and semi-quantitative PCR that detects all four human malaria species [43] (data not shown).

As described above, we could not detect any skewed distribution of the major subtypes of CD4 T cells between rural African (malaria exposed) and urban African individuals (not exposed), suggesting that endemic exposure to malaria does not affect the global CD4 T cell maturation profile in peripheral blood. This observation is consistent with a very recent study showing that terminally differentiated CD4 T cells, defined as CD45RA^+^ CD62L^−^, represented less than 1% of the total CD4 T cell pool of both healthy children and adults living in a malaria holoendemic region in western Kenya [44].

Although we found no evidence of “exhausted” CD4 T cells based on our panel of cell surface markers, more detailed and larger studies of *P. falciparum*-specific CD4 T cells in immune adults and children presenting with different severity of manifestations of malaria infection are required to analyse how endemic exposure and/or infection by *P. falciparum* may impact on the phenotypic characteristics of CD4 T cell compartment, including PD-1 and other markers [24], [45].

**Figure 6:**
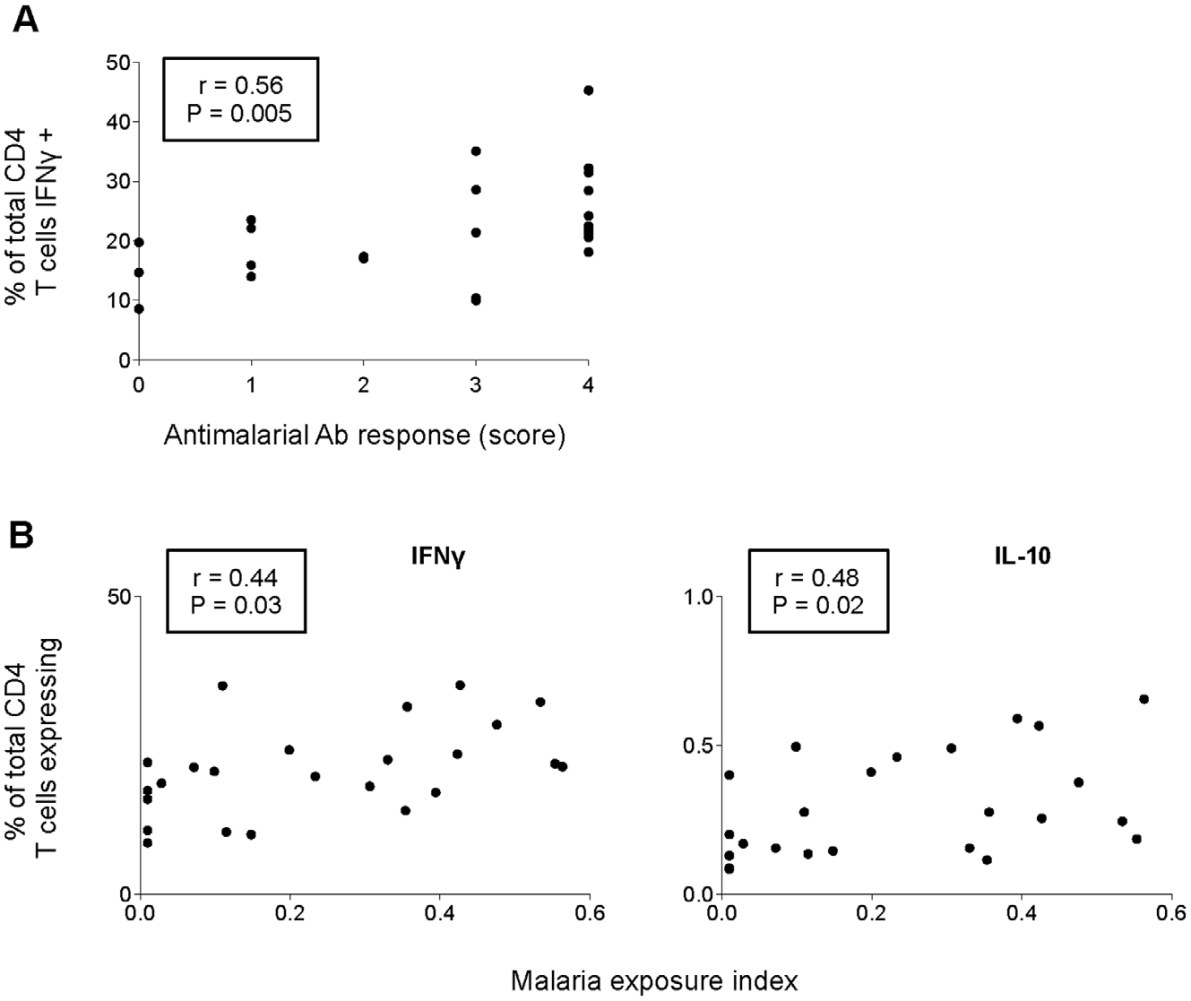
Total frequency of IFNγ and/or IL-10 expressing CD4 T cells correlates with malaria exposure. The percentage of total CD4 T cells positive for either IFNγ or IL-10 is plotted against either antimalarial Ab response, expressed by the number of Ag for which the Ab reactivity was defined as “high” levels (score between 0 and 4) (A), or malaria exposure indexes (B). Spearman’s correlation coefficients r and *P*-values are indicated for each plot.

To explore the relationship between the ability of CD4 T cells to produce specific cytokines and malaria exposure, correlation between the frequency of each cytokine response and antimalarial Ab levels or malaria exposure indexes was assessed by calculating the Spearman’s correlation coefficient. Interestingly, among the exposed donors, only the frequency of IFNγ producing CD4 T cells varied significantly with both antimalarial Ab levels (Spearman r = 0.56, P = 0.005) (Figure 6A) and malaria exposure indexes (Spearman r = 0.44, P = 0.03) (Figure 6B). This is in complete agreement with longstanding evidence for a strong bias towards a “Th1” response associated with malaria infection and the idea that this cytokine may be a biomarker of recent exposure and/or clinical immunity to malaria [46], although it is likely to be the case for many infections triggering this type of response. Likewise, a positive correlation was found between the frequency of IL-10 producing CD4 T cells and malaria exposure indexes (Spearman r = 0.48, P = 0.02) (Figure 6B); however, no correlation was found with *P. falciparum*-specific Ab levels (data not shown). Although we will need to characterize the functions of *P. falciparum*-specific CD4 T cell responses, this is consistent with a previous study reporting high frequencies of IL-10 expressing CD4 T cells in children with uncomplicated malaria [47]. Nevertheless it is similarly possibly the case after exposure to many infections, explaining in part why the correlation observed was weakly significant. No correlation was found between malaria exposure and CD4 T cell expression of any other cytokines (data not shown).

IL-10 producing CD4 T cells were found more frequent in rural donors and a certain proportion also co-expressed IFNγ, whereas IFNγ/IL-10 double producers were poorly represented in the CD4 T cell response from urban African donors and none was detectable within the CD4 T cells from European donors (Figure 4A and S5A). In addition, rural African individuals had a significantly decreased proportion of IFNγ single producers, compared to the urban volunteers (P = 0.009; P = 0.0005 when compared to UA and UE, respectively) (Figure S3A and S5A). When we then analysed the possible effect of malaria endemic exposure on the quality of IFNγ and IL-10 response, there was a trend towards a positive correlation between the relative frequency of CD4 T cell subset co-expressing IFNγ and IL-10 and malaria exposure indexes (Spearman r = 0.48, P = 0.017, Figure S5B), while we found no significant correlation with antimalarial Ab levels (data not shown). This finding was particularly notable as this CD4 T cell subset has previously been observed in populations living in malaria endemic areas after polyclonal stimulation with mitogen [47] or malaria-Ag specific activation of T cells [48], and we have shown that IL-10 production by IFNγ secreting CD4 T cells is a critical component of protection against severe malaria immunopathology in the mouse model of *P. chabaudi* infection [49]. All together, these results suggest that malaria endemic exposure, among other infections [50], may drive the expansion of this specific population of IFNγ^+^/IL-10^+^ CD4 T cells and this should be addressed in a larger study. By contrast, the relative frequency of IFNγ single expressing CD4 T cells was inversely correlated with the level of malaria exposure, estimated by exposure indexes (Spearman r = −0.47, P = 0.02, Figure S5B) but not with antimalarial Ab levels (data not shown). Of note, no direct correlation was found between Ab levels of any specificity and exposure indexes (data not shown). Acquisition and maintenance of antimalarial Ab depend on exposure to malaria infection; however, in adults who have acquired a significant degree of immunity to the infection, they are usually maintained even if parasite prevalence has fallen [51], which might partially explain the discrepancies observed.

Finally, no significant correlation was found between any other subset expressing IFNγ (shown in Figure 4) and malaria exposure (data not shown). Similarly, the relative frequency of multifunctional T cells (Figure 5) did not vary significantly with malaria exposure (i.e. no significant correlation was found with either antimalarial Ab levels or exposure indexes) (data not shown).

All together, we found only very little effect of malaria endemic exposure on the global phenotypic and functional attributes of CD4 T cell population in peripheral blood. Even though larger studies might be needed to show a significant effect, this is not surprising as these individuals are probably exposed to a myriad of microbial antigens hence increasing the memory pool to these pathogens while endemic malaria exposure drives or limits the expansion of only few discrete subpopulations of CD4 T cells. This is in complete agreement with growing body of evidence suggesting that multiple combinations of functions can be identified following polyclonal stimulation of CD4 T cells but Ag-specific CD4 T cell responses seem to be generally restricted to some particular subsets [52], [53].

### Conclusion

We have developed reliable and feasible assays, which allow us to draw the phenotypic profile as well as a very detailed picture of CD4 T cell cytokine responses, reflecting the global functional potential of CD4 T cell compartment in humans. Despite the small sample size in this pilot study, these assays revealed that there were no major differences in the distribution of CD4 T cell activation and memory phenotypes but that “Th1” type of cytokine profiles differed the most between rural and urban African individuals. With the caution that our assays might not be sensitive enough to fully characterize “Th2” type of response, there was a predominance of “Th1” over “Th2” type of cytokines in the global CD4 T cell cytokine expression profile of the rural community. The best quality, i.e. the highest number of subsets exhibiting distinct cytokine combinations, was found for this type of response. This observation was particularly interesting given long-lasting evidence for a strong bias towards a “Th1” response induced by many pathogens. In addition, rural African donors had 22% (mean of the overall analysis using the 3 Ab panels) of their cytokine secreting CD4 T cell population represented by multifunctional T cells, as opposed to 15% and 10% found for the urban African and European individuals, respectively. Our observations indicate that environmentally triggered T cell stimulation in rural Kenya leads to higher magnitude of cytokine response as well as an increase in multifunctional cells within the total CD4 T cell pool, while the presence of a dominant single cytokine producing CD4 T cell population seems associated with differential Ag experience in urban Kenya. CD4 T cell responses from the rural community were similar to those typically observed in the models of viral infections with Ag persistence but low levels of Ag exposure [9], whereas the predominance of single cytokine producing cells was associated with the presence of persistent and high levels of viral Ag [9]. Our observations do not support the general idea that high prevalence of chronic infectious diseases in developing countries causes persistent immune activation leading to hyporesponsiveness and anergy of T cell compartment [32], [33]. However, our results fit well the observations done with the model of *Leishmania major* infection, in which the persistence of low level infection or repeated exposure to Ag seem required to maintain a high frequency of multifunctional CD4 T cells [34], [54], [55]. Collectively, these data support the idea that the functionality of CD4 T cell response seems to be influenced by the conditions of Ag exposure and persistence and that the quality of CD4 T cell response may represent a proxy marker of environmentally driven T cell activation and Ag stimulation.

The associations between exposure to malaria and the relative frequency of some IFNγ-secreting CD4 T cell subsets suggest that malaria exposure could drive or limit the expansion of some discrete CD4 T cell subpopulations with specific functional profiles as a possible mechanism to control the infection or prevent immunopathology. The next step would be to analyse cytokine profiles of *Plasmodium*-specific CD4 T cells in malaria endemic populations in the context of larger longitudinal studies in order to link these functional signatures to protection or immunopathology associated with the infection.

## Materials and Methods

### Study Participants

This study took place in the Kenyan coastal region at KEMRI/Wellcome Trust Research Programme (WTRP) centre in Kilifi. Donors were enrolled after providing individual written informed consent. The rural population lives in the Junju sublocation of Kilifi district, a remote rural area, sparsely populated with limited infrastructure and poor housing. Inhabitants are predominantly farmers, commonly maintaining domestic animals and living mainly in stick and mud-built houses surrounded by agricultural lands [18]. 25 adults from this area were enrolled in the study. The mean age of the donors was 30 years (range: 20–50) and 72% were female. 16 age- and sex-matched African (n = 8) and European (n = 8) individuals, living in the urban area of Kilifi town, were used for comparison. Kilifi town is a coastal urban centre lying along the shores of the Indian Ocean. The urban African volunteers were all middle class Kenyans working at KEMRI/WTRP. European individuals were living in Kilifi at the time of the study but grew up and spent most of their life in urban areas in Europe before moving to Kenya. Several socio-economical studies comparing a rural and an urban area in Kilifi district in Coastal Kenya have shown disparities in terms of economical and social environments and personal development between the two areas and identified significantly higher levels of reported chronic and acute conditions in the rural as compared with the urban setting and differences in treatment-seeking patterns between settings [26], [56].

Junju is a stable malaria endemic area with two annual transmission seasons (from May to August and from October to December) and a parasite prevalence of 30% [18]. Samples were collected in a post-transmission season cross-sectional survey from December 2010 to February 2011. Absence of detectable parasitaemia at the time of sampling was checked for every donor with slide microscopy and confirmed by semi-quantitative PCR that detects all four human malaria species [43]. None of the urban volunteers has been exposed to malaria.

### Ethics Statement

The study was approved by the Kenyan Medical Research Institute, Ethical Review Committee under the SSC protocol number 1131, the Oxford Tropical Ethics Committee, protocol 030-06 as well as the National Research Ethics Service Committee at the MRC National Institute for Medical Research, reference 08/H0723/94.

### Parasite and *P. falciparum* Schizont Extract Preparation

*P. falciparum* parasites from the 2B2 strain were cultured *in vitro* using the method adapted from Trager *et al* [57]. The 2B2 strain is a clone derived from the laboratory strain ITO4 [58], [59]. Parasites were tested weekly for the absence of mycoplasma contamination by PCR following procedure described in [60]. Schizont-infected erythrocytes were isolated from sorbitol-synchronized cultures with magnet-activated cell sorter (MACS, Miltenyi). *Pf*SE was prepared by two rapid freeze-thaw cycles in liquid nitrogen and a 37°C water bath. Extracts of uninfected erythrocytes were prepared similarly.

### Recombinant Antigens

*P. falciparum*-specific IgG responses were quantified against a set of recombinant antigens including *P. falciparum* AMA-1 (AMA-1-FVO/3D7), MSP-1 42 kDa and MSP-2 (MSP-2_CH150/9). AMA-1 and MSP-1 were histidine-tagged fusion proteins kindly given by Dr Francis Ndungu (KEMRI, Kilifi) [61], while MSP-2 was a glutathione S-transferase (GST) fusion protein kindly provided by Dr Faith Osier (KEMRI, Kilifi) [39].

### Determination of *P. falciparum-*specific IgG by ELISA

Whole blood samples were first centrifuged at 1500 g for 10 minutes and plasmas were collected and kept at −80°C for each individual. Plasma levels of immunoglobulin G (IgG) specific to *Pf*SE as well as AMA-1, MSP-1 and MSP-2 recombinant antigens were determined by enzyme-linked immunosorbent assays (ELISA), as previously described [61]. We confirmed that none of the plasmas was reacting to uninfected erythrocyte lysate or GST tag alone using the same procedure. The background reactivity against these negative controls was subtracted from the OD values for the corresponding antigen of interest. For AMA-1 and MSP-1 antigens, the mean OD value of sample diluent buffer alone was subtracted. Plasmas were tested in duplicate (diluted from 1/250 up to 1/32000 by 8 serial ½ dilutions) in the presence of two negative control plasma samples and a sample of pooled non-immune sera obtained from European donors who have never been exposed to malaria as well as two positive control samples and a sample of pooled African immune sera, known to have high antimalarial Ab concentration. An OD value above the mean OD value obtained for each antigen with the negative plasma samples +/−3 standard deviations defined a positive Ab response. Median OD values were used as thresholds (cut-offs) to characterise IgG Ab reactivity as “high” versus “low” levels for each antigen. A system of score, from 0 to 4, of how many Ag were recognised above the cut-offs was then applied to each individual, where 0 indicates low/undetectable Ab to all 4 tested Ag and 1, 2, 3, 4 denote high levels of Ab to 1, 2, 3 or all 4 Ag, respectively. Of note, antimalarial Ab levels of the different specificities were significantly correlated with one another and, for most of them, there was also a significant correlation with age (data not shown).

### Calculation of Malaria Exposure Indexes for Malaria-exposed Individuals

Malaria exposure indexes were calculated, as described in [42], as an estimate of individual’s exposure to the infection. They represent the distance-weighted local prevalence of malaria infection, i.e. the proportion of individuals with asymptomatic or symptomatic parasitaemia, within 1 km radius of each indexed adult between 2009 and 2010, just before the bleed. Clinical and cross sectional data, from active malaria surveillance in the field, from 850 individuals were used to estimate the exposure index of each individual.

### PBMC Stimulation

PBMC were distributed in 96-well round-bottom plates at 0.5×10^6^/well. Cells were cultured in RPMI medium (RPMI 1640; Gibco) supplemented with L-glutamine (2 mM; Gibco), penicillin/streptomycin (10%; Gibco), fœtal calf serum (10%; Sigma), sodium pyruvate (0.5 mM; Gibco) and Hepes buffer (6 mM; Gibco). PBMC were stimulated in the presence of phorbol 12,13-dibutyrate (PdBU, 50 mM; Sigma) and ionomycin (500 ng/ml; Sigma) for 6h at 37°C in 5% CO2. Of note, PdBU can be substituted by phorbol 12-myristate 13-acetate (PMA) for PBMC activation. For intracellular cytokine assays, brefeldin A (10 µg/ml; Sigma) was added after 2 h of activation during the last 4 h of culture to inhibit protein secretion. Following 6 h stimulation, cells were harvested, fixed, stained and analysed as described below.

### Antibodies and Reagents

FITC-CD3 (clone OKT3), PE-CD45RA (clone HI100), PerCPCy5.5-CD28 (clone CD28.2), PECy7-CD4 (clone RPA-T4), APC-CD197 (CCR7) (clone TG8/CCR7), APCCy7-CD27 (clone O323), Pacific Blue-CD14 (clone HCD14), PE-IL-10 (clone JES3-9D7), PerCPCy5.5-IL-17 (clone BL168), APC-TNFα (clone mAb11), APCCy7-CD69 (clone FN50), PE-IL-21 (clone 3A3-N2), PE-IL-9 (clone MH9A4), APC-IL-4 (clone 8D4-8) and PECy7-IL-10 (clone JES3-9D7) monoclonal Ab (mAb) as well as APC mouse IgG2a, APCCy7 mIgG1, FITC RatIgG2a, PE mIgG1, PE mIgG2b, PE RatIgG1, PerCPCy5.5 mIgG1, PECy7 mIgG1 and PECy7 RatIgG1 isotype control Ab were from Biolegend, Cambridge BioScience. PE/Texas Red-CD4 mAb (clone RFT-4g) was from Southern Biotech, Cambridge BioScience. PECy7-IFNγ mAb (clone 4S.B3) and APC mouse IgG1 isotype control Ab were purchased from BD Biosciences. FITC-IL-2 (clone MQ1-17H12) mAb was from eBioscience, Insight Biotechnology, London, UK. APC-IL-22 mAb (clone 142928) was from R&D System. The reagent panels used in this study were developed following procedures detailed in [62] and are presented in Table S1. We titrated each antibody and the final dilution was selected for an optimal specific staining associated with a low background using the corresponding isotype control antibody.

### Flow Cytometry Analysis

PBMC were isolated from heparinized blood by Ficoll/Paque™ Plus (GE Healthcare) centrifugation and distributed in 96-well round-bottom plates at 0.5×10^6^/well. Cells were stained for 30min at 4°C with appropriate combinations of fluorochrome conjugated mAb in the presence of 5% normal mouse serum (NMS) and fixed in 2% paraformaldehyde (Sigma) in PBS. For intracellular staining, fixed cells were permeabilized and stained in saponin (0.5% in PBS; Sigma) with the different panels of Ab specific for the cytokines of interest as described in Table S1, in the presence of 10% NMS. For the determination of CD4 T cell differentiation profile, phenotypes were assessed directly *ex vivo* on fresh unstimulated cells and we excluded markers, such as CD62L, whose expression can be lost with cryopreservation [63] in order to be able to apply our assays to cryopreserved samples. Surface staining was thus performed on freshly isolated PBMC and at least 200,000 lymphocytes were acquired using forward scatter (FSC)/side scatter (SSC) gate. T cell maturation phenotypes were defined by the differential expression of the cell surface markers CCR7, CD45RA, CD28 and CD27 [4], [16]. The markers for the discrimination of the distinct phenotypes were set using isotype control mAb to define the threshold of positivity for each marker. Figure S1 shows the gating strategy for this analysis. For CD4 T cell cytokine response analysis, PBMC were stimulated non-specifically with PdBU and ionomycin as described above and the production of IFNγ, IL-2, IL-10, IL-17, TNFα, IL-21, IL-22, IL-4 and IL-9 by CD4 T cells was evaluated using three different panels of Ab (panels 1–3, Table S1), measuring independently but simultaneously up to 5 functions. Panels 1 and 2 defined “Th1/Th17” types of cytokine expression profiles while panel 3 analysed “Th2/Th9” types of cytokine secretion profiles. Surface staining was performed on activated cells and then followed by intracellular staining. At least, 35,000 events in the CD4+ lymphocyte gate were analysed for the expression of cytokine. The gating scheme for this analysis is illustrated in Figure S2. During the subsequent FC analyses, CD4+ monocytes were excluded from the CD4 T cell gate by costaining cells with the monocyte marker CD14. Non-specific background was determined using isotype matched control mAb and was subtracted from all the cytokine measurements. Specific cytokine staining was then confirmed by concomitant cell-surface staining with CD69 as a marker of activated lymphocytes. Only cells clearly positive for CD69 were considered as cytokine producing cells. Within CD69 positive CD4 T cells, the subset of cells expressing each cytokine was determined. Cells were analysed using a 9-colour CyAn ADP flow cytometer (Beckman Coulter) and the data analysis was performed using FlowJo software version 8.8.7 (TreeStar). For the phenotypic analysis of CD4 T cell differentiation subsets or to define the quality of CD4 T cell cytokine response, the subset of cells that express each functional marker within the CD4 T cell population was entered into a ‘Boolean gating’ analysis [13] that separately identifies all the subpopulations expressing each possible combination of markers or functions. The frequency of each specific CD4 T cell subpopulation was expressed as a percentage of CD4 T cells (%). Similarly, the magnitude of the total cytokine response as well as the frequency of CD4 T cells producing each particular cytokine was represented as a percentage of CD4 T cells (%). To analyse the pattern of the responses i.e. the relative frequency of the different cytokines within the overall cytokine producing CD4 T cell population or when CD4 T cell subsets were grouped according to the number of cytokines they simultaneously expressed, the data were normalized as follow. The total number of CD4 T cells positive for at least one cytokine was considered as 100%. The frequency of each CD4 T cell subpopulation was then expressed as a fraction of the overall cytokine response by CD4 T cells (%) by dividing each value by the sum of all the responses with at least one positive cytokine and multiplying by 100. For the functional profiling of CD4 T cell cytokine response, only responses with more than 10 positive events for cytokine production and a magnitude more than 3 times that of the background staining with isotype controls were considered.

### Statistical Analysis

Statistical analysis was performed in GraphPad Prism software version 5.0d. Across group comparisons were performed using Kruskal-Wallis test. When the medians were found to vary significantly between groups (P<0.05), nonparametric Mann-Whitney U test was used to analyse differences in the T cell responses between rural and urban individuals, by pair-wise comparisons of unpaired samples. Bivariate correlations between CD4 T cell responses and *P. falciparum*-specific Ab levels or malaria exposure indexes were performed using Spearman rank nonparametric correlation test. All tests were 2-tailed, with P<0.05 considered statistically significant. *indicates P<0.05, **P≤0.01 and ***P≤0.001, respectively. No adjustment was made for multiple comparisons because this study was by nature exploratory.

## Supporting Information

Figure S1 **Gating strategy for polychromatic flow cytometry analysis of CD4 T cell differentiation profile.** Initial gating was performed on lymphocytes and singlets only were included in the analysis. CD4 T cells were then gated based on the concomitant expression of CD3 and CD4. Within the CD4 T cell population, the subset of cells expressing each marker was determined. Isotype matched control mAb were used to set up the threshold of positivity for each marker as shown below each plot. The plots shown are representative of all the analysed samples.
(TIF)

Figure S2 **Gating strategy for CD4 T cell cytokine production analysis.** The analysis was initially gated on lymphocytes and only singlets were included. CD4 T cells were then gated based on both the expression of CD4 and the lack of expression of the monocyte marker CD14. Within the CD4 T cell population, only cells clearly positive for CD69 were considered as cytokine producing cells and included in the analysis. Within the CD4 T cells CD69 positive, the subset expressing each cytokine of interest (IFNγ, IL-2, IL-10, IL-17, TNFα, IL-21, IL-22, IL-4 and IL-9, respectively) was defined. Non-specific background was determined using isotype matched control Ab as shown below each plot for cytokine staining and was subtracted from all the data. The plots shown are representative of all the analysed samples.
(TIF)

Figure S3 **Functional characterization of CD4 T cell cytokine response by Boolean gating analysis.** The composition of the CD4 T cell cytokine responses from each group of donors was analysed using three different panels of Ab (panel 1–3 Table S1). For simplicity, only the individual combinations of cytokines observed in all three groups are shown. The contribution of the indicated functional response (x-axis) toward the total CD4 T cell cytokine response is expressed as a percentage (medians and 95% confidence intervals are represented) and compared between each group of donors (rural African donors (RA, n = 25); urban African (UA, n = 8) and European donors (UE, n = 8)). The cytokine combinations are indicated in the panel below each plot. Each dot denotes positivity for each cytokine indicated on the left. A shows the data obtained with panel 1, while B and C represent the results obtained with panel 2 and 3, respectively. Differences in the relative frequency of each CD4 T cell subset across groups were tested using Kruskal-Wallis test (data not shown) and where significance was obtained, Mann-Whitney U test was used for pair-wise analysis between groups. Significant differences are indicated by an asterisk. *indicates P<0.05, **P≤0.01 and ***P≤0.001, respectively.
(TIF)

Figure S4 **Specific IgG Ab responses to malarial antigens.** Levels of antimalarial serum IgG were determined by antigen specific ELISA. Ab levels are expressed by the OD values obtained with the sera dilution 1/500 and shown for each tested *P. falciparum* antigen i.e. total schizont extracts (*Pf*SE), AMA-1, MSP-1 and MSP-2, respectively. Shown is the comparison of Ab levels between rural African donors (RA, black circles), endemically exposed to malaria, and urban unexposed African (UA, black squares) and European donors (UE, black triangles). Bars indicate mean +/−95% confidence intervals. Mann-Whitney U test was used for pair-wise analysis of the differences in Ab levels between the rural African individuals and each urban group. Statistically significant *P*-values (<0.05) are indicated by an asterisk(****P≤0.0001).
(TIF)

Figure S5 **Endemic exposure to malaria influences IFN**γ **and IL-10 expression profile by CD4 T cells.** (A) CD4 T cell responses from each group of donors were analysed using panel 1 (Table S1) as shown in more detail in Figure S3A. For simplicity, here is only represented the pattern of IFNγ and IL-10 production by CD4 T cells. The relative proportion of CD4 T cells producing each combination of cytokines (shown in the panel on the right) is represented as a percentage of the total CD4 T cell IFNγ and IL-10 response (mean with SEM are shown for each group) and was compared between the 3 groups of donors (rural African (RA), urban African (UA) and urban European donors (UE)). Each dot denotes the expression of IFNγ or IL-10 as indicated on the left. CD4 T cell subsets were then ordered according to the number of cytokines they expressed, as specified by the horizontal bars of different colours showing these combinations of 2 or 1 cytokine. B shows the trend towards a negative and a positive correlation between malaria exposure and the relative frequencies of IFNγ single producers (SP) and IFNγ/IL-10 double producers (DP), respectively. The relative frequency of IFNγ producing CD4 T cells expressing IFNγ alone or in combination with IL-10 is plotted against malaria exposure indexes. Spearman’s correlation coefficients r and *P*-values are indicated for each plot.
(TIF)

Table S1 **Panels for phenotypic and functional characterization of CD4 T cell population by polychromatic flow cytometry.** FITC, fluorescein; PE, R-phycoerythrin; PerCP, peridinin chlorophyll protein; Cy, cyanine; APC, allophycocyanin.
(TIF)
