# Supplementary figures and images for: Phenotypic and Functional Profiling of CD4 T Cell Compartment in Distinct Populations of Healthy Adults with Different Antigenic Exposure
Source: PLoS One. 2013 Jan 28;8(1):e55195. doi: 10.1371/journal.pone.0055195 (PMC3557244; doi:10.1371/journal.pone.0055195)

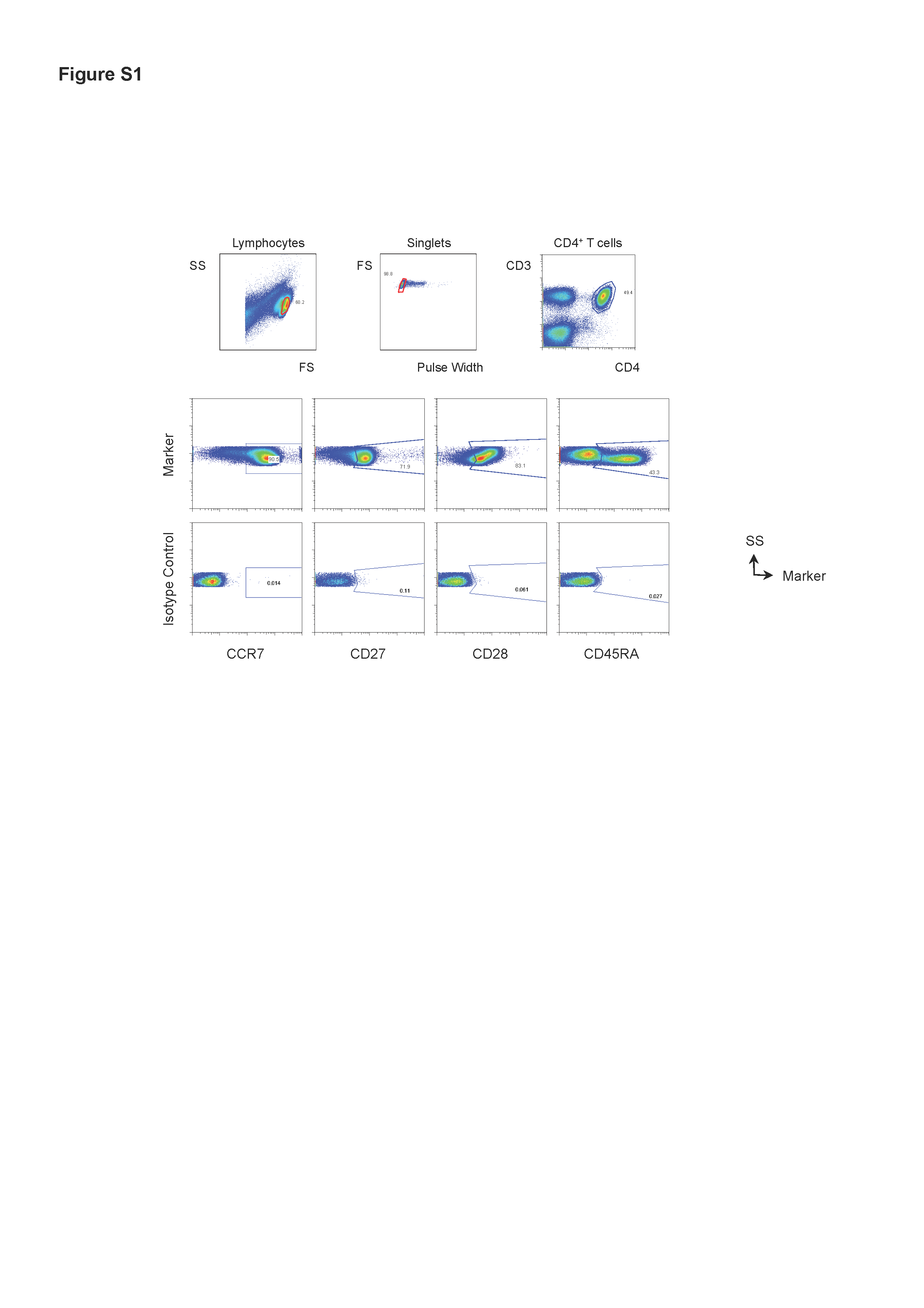

Supplement: Figure S1 — Gating strategy for polychromatic flow cytometry analysis of CD4 T cell differentiation profile. Initial gating was performed on lymphocytes and singlets only were included in the analysis. CD4 T cells were then gated based on the concomitant expression of CD3 and CD4. Within the CD4 T cell population, the subset of cells expressing each marker was determined. Isotype matched control mAb were used to set up the threshold of positivity for each marker as shown below each plot. The plots shown are representative of all the analysed samples. (TIF) [file pone.0055195.s001.tif]

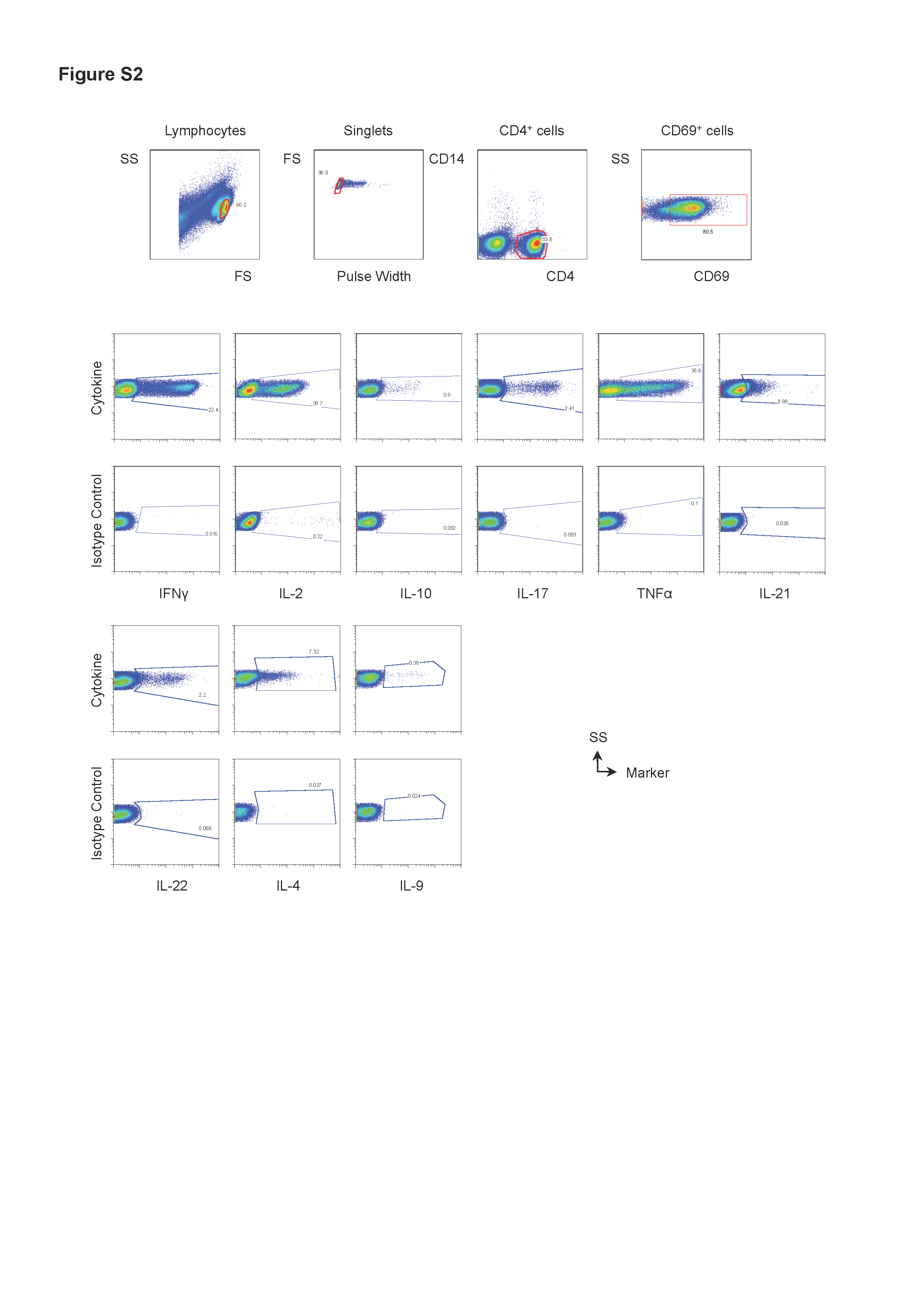

Supplement: Figure S2 — Gating strategy for CD4 T cell cytokine production analysis. The analysis was initially gated on lymphocytes and only singlets were included. CD4 T cells were then gated based on both the expression of CD4 and the lack of expression of the monocyte marker CD14. Within the CD4 T cell population, only cells clearly positive for CD69 were considered as cytokine producing cells and included in the analysis. Within the CD4 T cells CD69 positive, the subset expressing each cytokine of interest (IFNγ, IL-2, IL-10, IL-17, TNFα, IL-21, IL-22, IL-4 and IL-9, respectively) was defined. Non-specific background was determined using isotype matched control Ab as shown below each plot for cytokine staining and was subtracted from all the data. The plots shown are representative of all the analysed samples. (TIF) [file pone.0055195.s002.tif]

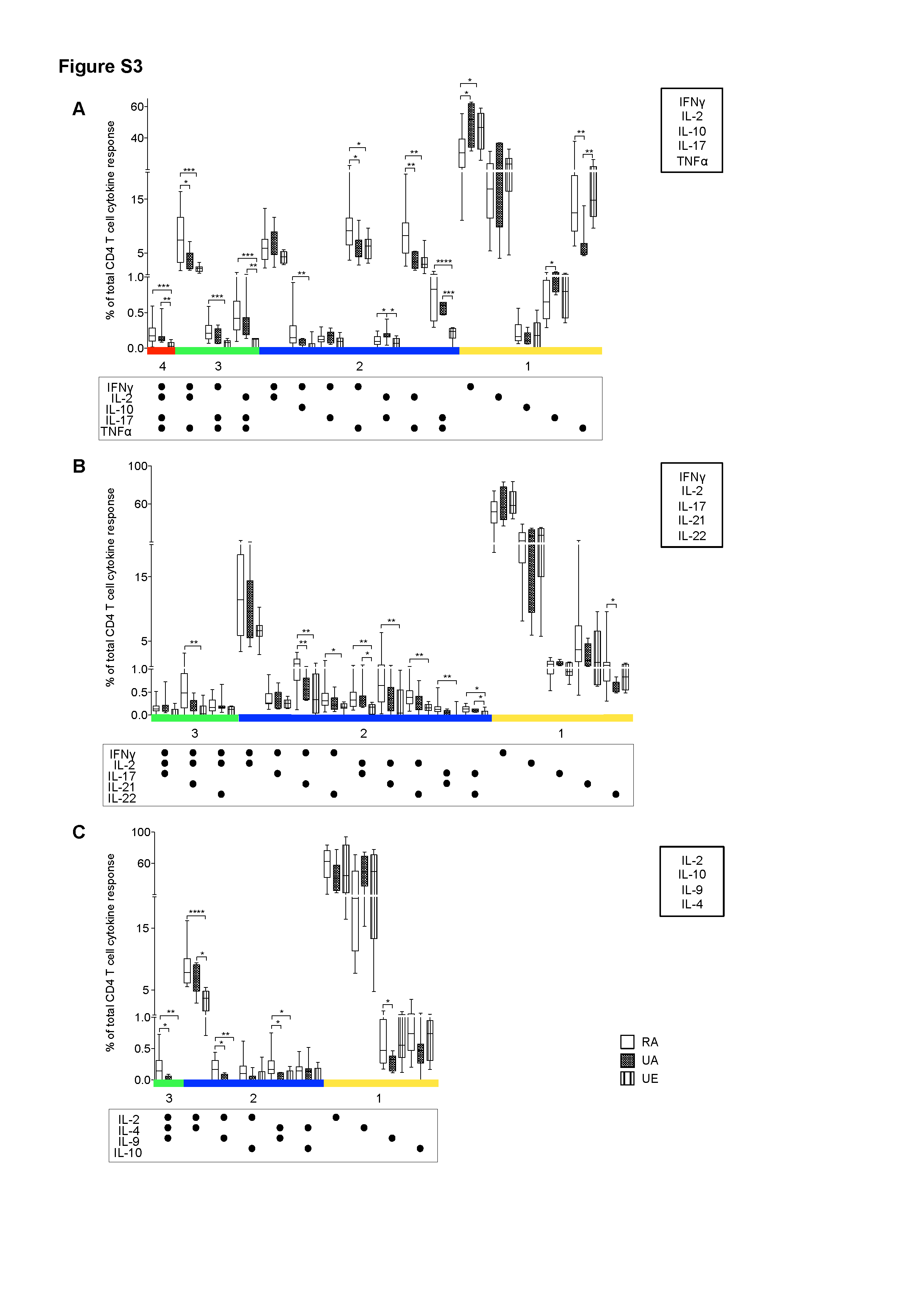

Supplement: Figure S3 — Functional characterization of CD4 T cell cytokine response by Boolean gating analysis. The composition of the CD4 T cell cytokine responses from each group of donors was analysed using three different panels of Ab (panel 1–3 Table S1). For simplicity, only the individual combinations of cytokines observed in all three groups are shown. The contribution of the indicated functional response (x-axis) toward the total CD4 T cell cytokine response is expressed as a percentage (medians and 95% confidence intervals are represented) and compared between each group of donors (rural African donors (RA, n = 25); urban African (UA, n = 8) and European donors (UE, n = 8)). The cytokine combinations are indicated in the panel below each plot. Each dot denotes positivity for each cytokine indicated on the left. A shows the data obtained with panel 1, while B and C represent the results obtained with panel 2 and 3, respectively. Differences in the relative frequency of each CD4 T cell subset across groups were tested using Kruskal-Wallis test (data not shown) and where significance was obtained, Mann-Whitney U test was used for pair-wise analysis between groups. Significant differences are indicated by an asterisk. *indicates P<0.05, **P≤0.01 and ***P≤0.001, respectively. (TIF) [file pone.0055195.s003.tif]

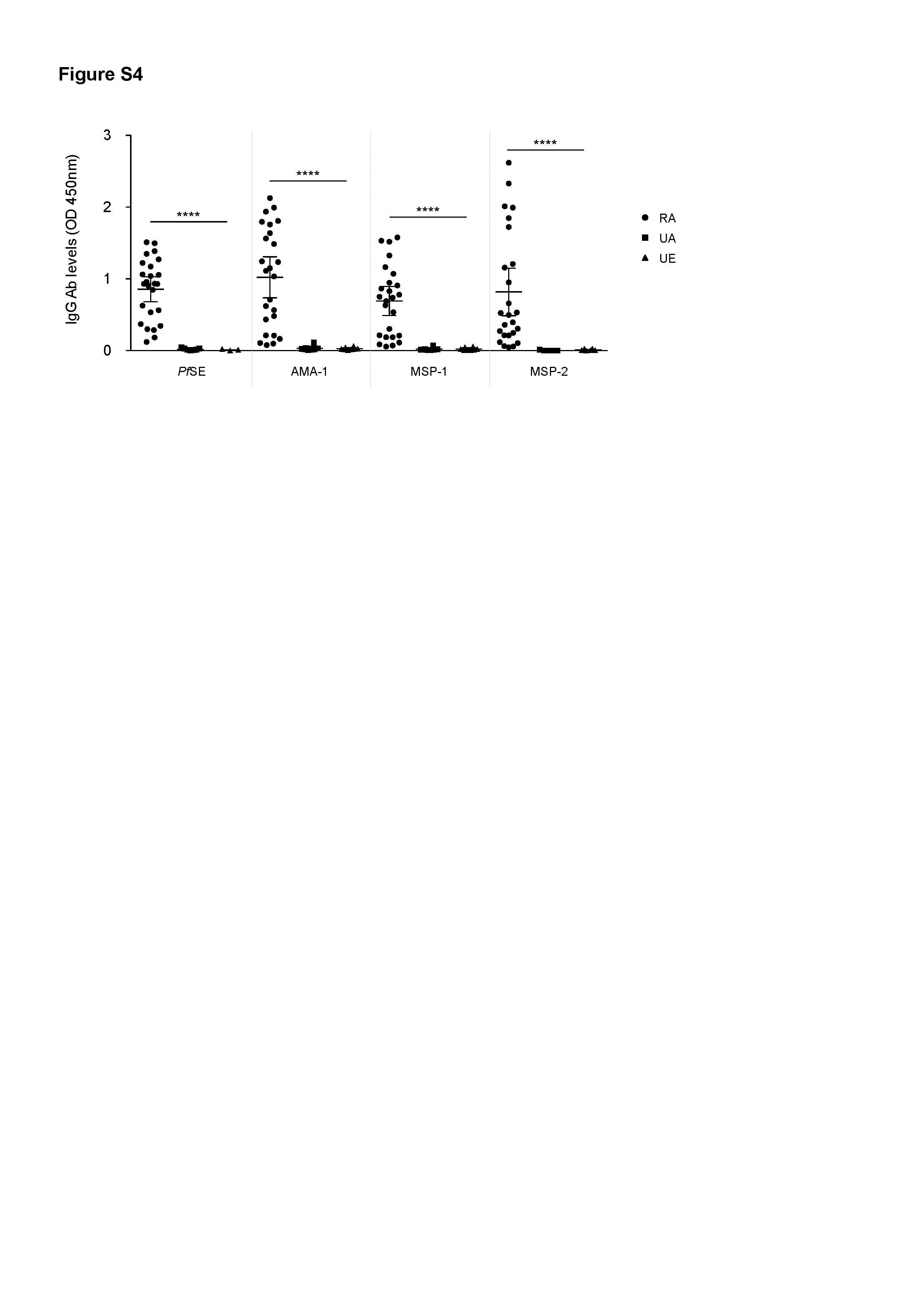

Supplement: Figure S4 — Specific IgG Ab responses to malarial antigens. Levels of antimalarial serum IgG were determined by antigen specific ELISA. Ab levels are expressed by the OD values obtained with the sera dilution 1/500 and shown for each tested P. falciparum antigen i.e. total schizont extracts (PfSE), AMA-1, MSP-1 and MSP-2, respectively. Shown is the comparison of Ab levels between rural African donors (RA, black circles), endemically exposed to malaria, and urban unexposed African (UA, black squares) and European donors (UE, black triangles). Bars indicate mean +/−95% confidence intervals. Mann-Whitney U test was used for pair-wise analysis of the differences in Ab levels between the rural African individuals and each urban group. Statistically significant P-values (<0.05) are indicated by an asterisk(****P≤0.0001). (TIF) [file pone.0055195.s004.tif]

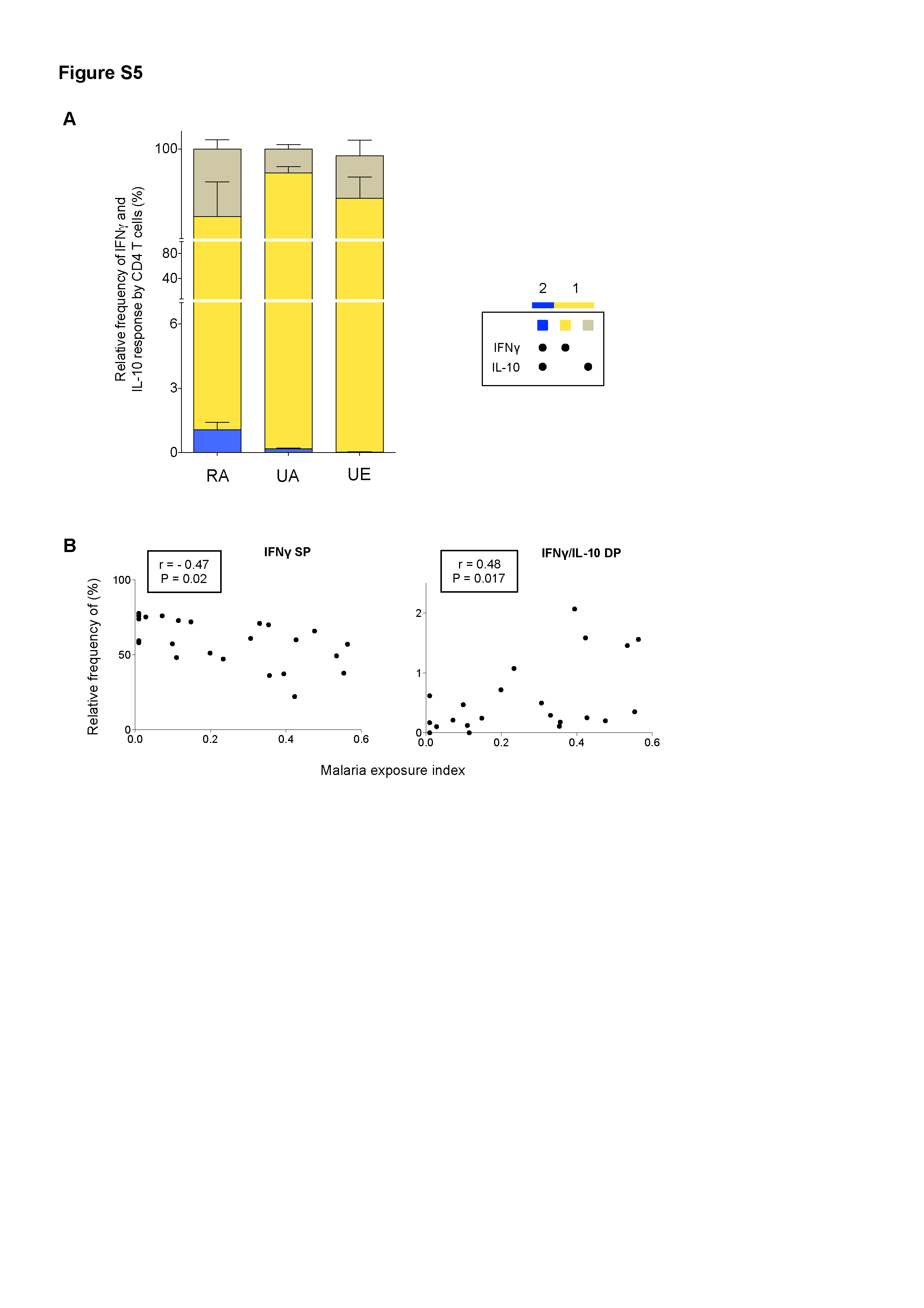

Supplement: Figure S5 — Endemic exposure to malaria influences IFNγ and IL-10 expression profile by CD4 T cells. (A) CD4 T cell responses from each group of donors were analysed using panel 1 (Table S1) as shown in more detail in Figure S3A. For simplicity, here is only represented the pattern of IFNγ and IL-10 production by CD4 T cells. The relative proportion of CD4 T cells producing each combination of cytokines (shown in the panel on the right) is represented as a percentage of the total CD4 T cell IFNγ and IL-10 response (mean with SEM are shown for each group) and was compared between the 3 groups of donors (rural African (RA), urban African (UA) and urban European donors (UE)). Each dot denotes the expression of IFNγ or IL-10 as indicated on the left. CD4 T cell subsets were then ordered according to the number of cytokines they expressed, as specified by the horizontal bars of different colours showing these combinations of 2 or 1 cytokine. B shows the trend towards a negative and a positive correlation between malaria exposure and the relative frequencies of IFNγ single producers (SP) and IFNγ/IL-10 double producers (DP), respectively. The relative frequency of IFNγ producing CD4 T cells expressing IFNγ alone or in combination with IL-10 is plotted against malaria exposure indexes. Spearman’s correlation coefficients r and P-values are indicated for each plot. (TIF) [file pone.0055195.s005.tif]

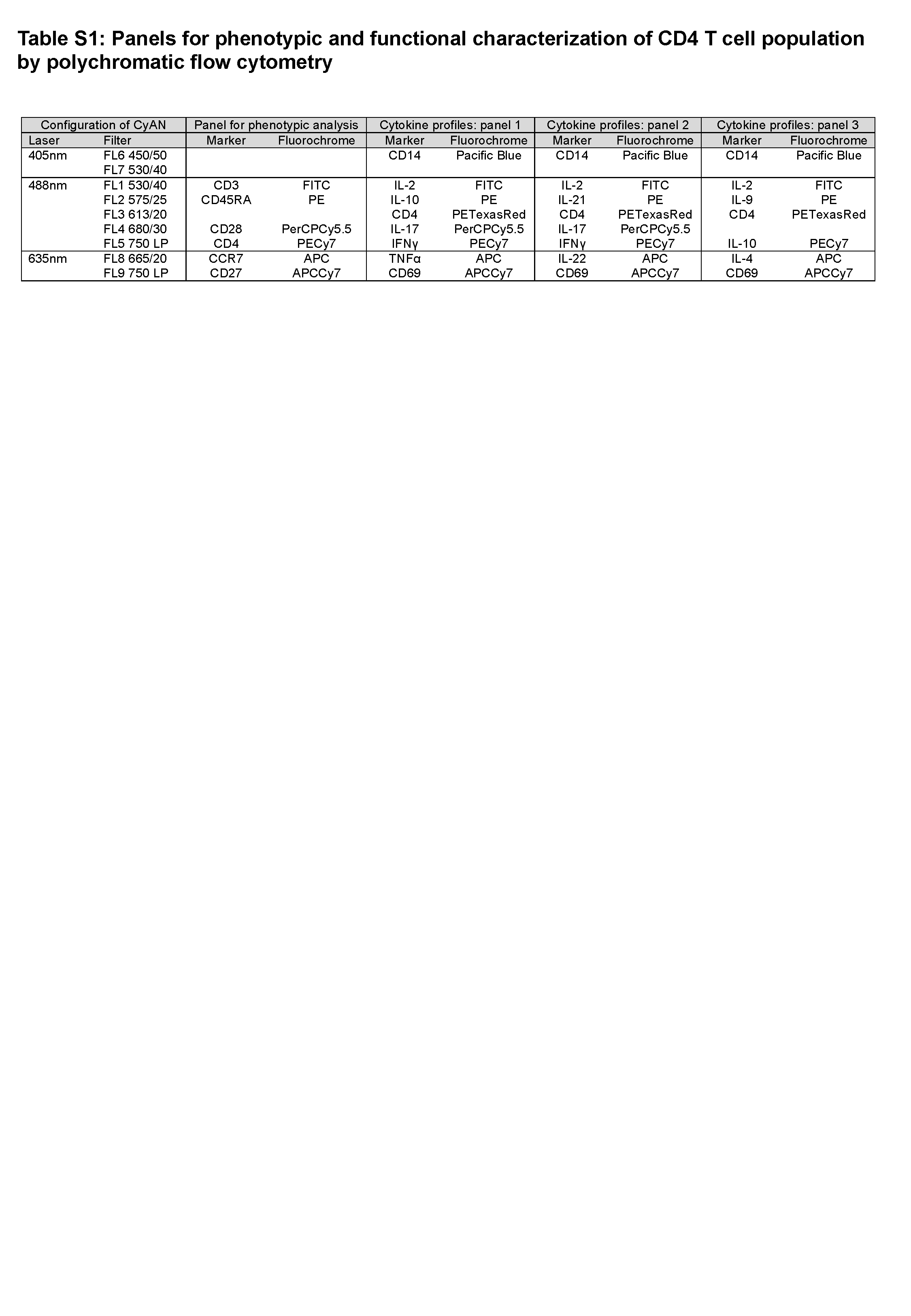

Supplement: Table S1 — Panels for phenotypic and functional characterization of CD4 T cell population by polychromatic flow cytometry. FITC, fluorescein; PE, R-phycoerythrin; PerCP, peridinin chlorophyll protein; Cy, cyanine; APC, allophycocyanin. (TIF) [file pone.0055195.s006.tif]
